# Mortality Prediction in Heart Failure Patients: Machine Learning Versus Get With The Guidelines-Heart Failure (GWTG-HF) and Acute Decompensated Heart Failure National Registry (ADHERE)

**DOI:** 10.7759/cureus.89226

**Published:** 2025-08-01

**Authors:** Dayanna Q Palmer, Ronaldo A Gismondi, Pedro Gemal, Guilherme S Lomba, Flavio L Seixas

**Affiliations:** 1 Internal Medicine, Universidade Federal Fluminense, Niterói, BRA; 2 Computer Science, Universidade Federal Fluminense, Niterói, BRA

**Keywords:** heart failure, in-hospital mortality, machine learning, mimic-iv database, risk prediction

## Abstract

Background

Predicting the mortality risk in Heart Failure (HF) patients is crucial for identifying high-risk individuals and establishing appropriate treatment strategies. Machine-learning algorithms offer immense potential for the risk prediction of cardiovascular conditions.

This study aimed to evaluate and compare the predictive performance of Extreme Gradient Boosting (XGB), a machine-learning algorithm, with two traditional scores, Acute Decompensated Heart Failure National Registry (ADHERE) and Get With The Guidelines-HF (GWTG-HF), for mortality risk in patients admitted to the Cardiac Intensive Care Unit (CICU) with HF.

Methods

Using data from the Medical Information Mart for Intensive Care IV (MIMIC-IV) database, we selected patients who were 18 years or older, with a diagnosis of HF admitted to CICU. We developed a risk prediction model using the XGB algorithm and computed the ADHERE and GWTG-HF scores for comparison. The predicted variable was the in-hospital mortality rate. The performance of these scores and the XGB model was assessed using the area under the receiver operating characteristic curve (AUC).

Results

A total of 5602 adult patients were included in the study and randomly divided into a derivation group (n = 4481, 80%) and a validation group (n = 1121, 20%) for analysis. The analysis revealed 346 (6.2%) in-hospital deaths. The performance of our XGB-based model (AUC = 72.1%, 95% CI: 65.23%-79%) was superior to that of GWTG-HF (AUC = 65.5%, 95% CI: 58.7%-72.2%) and ADHERE (AUC = 63.4%, 95% CI: 56.6%-70.2%).

Conclusion

The XGB-based model demonstrated superior performance to the ADHERE and GWTG-HF models, suggesting its utility for enhancing clinical decision-making. Routine implementation and evaluation in prospective studies are indicated to validate its potential in real-world settings.

## Introduction

Heart failure (HF) has an estimated prevalence of 64 million people globally, constituting approximately 2% of the adult population [1,2]. This condition imposes a significant global burden in terms of morbidity and mortality, with up to 83% of patients requiring hospitalization at least once [3]. HF mortality estimates vary significantly depending on the demographic characteristics of the study population. In a comprehensive systematic review of high-income countries, the 10-year survival rate was 34.9% [2,4].

Predicting the mortality risk in HF patients is crucial for identifying high-risk individuals and establishing appropriate treatment strategies [3]. Several risk scores have been developed for this purpose, such as Acute Decompensated Heart Failure National Registry (ADHERE) [5], Get With the Guidelines - Heart Failure (GWTG-HF) [6], Enhanced Feedback for Effective Cardiac Treatment (EFFECT) [7], and Organized Program to Initiate Lifesaving Treatment in Hospitalized Patients With Heart Failure (OPTIMIZE-HF) [8]. However, most of these scores do not perform sufficiently when applied to populations other than the original ones [9].

Machine-learning algorithms offer immense potential for the risk prediction of cardiovascular conditions, including HF. These algorithms employ computational techniques to analyze substantial volumes of data and detect concealed patterns or relationships that may not be readily discernible [10,11]. Nevertheless, uncertainties remain regarding the implications of these tools in clinical practice considering their pitfalls and potential biases [12]. In this context, the present study aimed to evaluate and compare the predictive performance of Extreme Gradient Boosting (XGB), a machine-learning algorithm, with two traditional scores (ADHERE and GWTG-HF) for in-hospital mortality risk in CICU-admitted HF patients, with the ultimate goal of supporting early risk stratification and improving critical care outcomes.

## Materials and methods

Data source and study population

The Medical Information Mart for Intensive Care IV (MIMIC-IV) dataset was used in this study. MIMIC is an open-source database that is composed of anonymized health data from over 70,000 Intensive Care Unit (ICU) admissions of patients aged 18 years and older at the Beth Israel Deaconess Medical Center in Boston, Massachusetts, United States of America, between 2008 and 2019. It comprises information on laboratory measures, microbiological tests, medication prescriptions, procedures, comorbidities, clinical notes from healthcare professionals, discharge summaries, radiology reports, and survival status [13].

After completing the National Institutes of Health online training and the Protecting Human Research Participants exam (no. 45395590), the researchers obtained permission to access the dataset from this project. The authors were responsible for the integrity of the data and the subsequent analyses. The requirement for individual patient consent was waived because the project did not affect clinical care, and all protected health information was anonymized. The study protocol was approved by the Ethics Committee of the Universidade Federal Fluminense (approval no. 60055422.8.00005243).

We selected patients who were 18 years or older, with a diagnosis of HF, identified by a review of the following International Classification of Diseases, Ninth Revision (ICD-9-CM) codes: 402.01, 402.11, 402.91, 404.01, 404.03, 404.11, 404.13, 404.91, 404.93, 428.02, 428.03, and 428.04, or with a diagnosis of respiratory failure identified by the following ICD-9-CM codes: 518.81, 518.82, and 518.84. Patients with invalid or incomplete records (missing heart rate measurements, incomplete administrative procedures, no record of admission or discharge from the ICU, and missing one of the variables used in the ADHERE or GWTG scores), organ donors, and patients with hospitalizations lasting less than four hours were excluded from the study.

Data extraction

Following MIMIC-IV guidelines, we used Structured Query Language (SQL) to access the dataset and extract variables. These included demographic characteristics (race, sex, age at admission, weight, and height) and vital signs, such as urine output over 24 hours, body temperature, diastolic blood pressure (DBP), systolic blood pressure (SBP), blood oxygen saturation, mean blood pressure (MBP), respiratory rate (RR), and heart rate (HR). We also collected data on prior comorbidities, including peripheral vascular disease, cerebrovascular disease, myocardial infarction, kidney disease, diabetes, and chronic obstructive pulmonary disease (COPD). Finally, laboratory measurements included N-terminal pro-B-type natriuretic peptide (NT-proBNP), creatine kinase, lactate, pH, pCO₂, pO₂, bicarbonate, anion gap, neutrophils, basophils, lymphocytes, hemoglobin, hematocrit, mean corpuscular hemoglobin concentration (MCHC), white blood cells (WBC), mean corpuscular volume (MCV), mean corpuscular hemoglobin (MCH), glucose, calcium, sodium, magnesium, potassium, chloride, creatinine, and blood urea nitrogen (BUN).

The demographic characteristics, vital signs, and laboratory measurements were defined as the first values recorded after or closest to the time of CICU admission. Race and ethnicity categories were presented as separate questions, following the standards set by the Office of Management and Budget (OMB) [14].

Laboratory measurements were defined as the most extreme measurement presented during the first 24 h after CICU admission, following clinical relevance (e.g., the maximum creatinine value in the first 24 h of admission was analyzed). Vital signs were measured multiple times (taken every five minutes), and the calculated arithmetic average was included in the analysis. Previous comorbidities were also analyzed.

The primary outcome was in-hospital mortality, which was defined as the vital status at the time of hospital discharge. This attribute was assessed using supervised learning.

Missing data handling

Missing data are a common problem in longitudinal datasets such as MIMIC-IV. Recognizing that the exclusion of patients might introduce bias into the study, we eliminated screening variables with missing values exceeding 25% [15]. For the remaining variables, missing values were imputed using the k-nearest neighbors (KNN) algorithm [16], specifying k = 10 to define the number of nearest neighbors considered during the imputation process. Continuous variables were standardized prior to imputation to ensure accurate distance calculations.

Data splitting and preprocessing

To construct the classification models, the outcome variable - in-hospital mortality - was treated as a binary variable (death or discharge). The dataset was randomly split into two subsets in a stratified manner: 80% of the data were used for model training and 20% for testing, ensuring that the proportion of mortality events was preserved across both sets [17]. All steps of data processing and modeling were implemented in Python, and the complete code is publicly available to ensure transparency and reproducibility (https://github.com/pedrogemal/mortality-prediction-in-hf).

ADHERE and GWTG risk scores

ADHERE and GWTG predict in-hospital mortality in patients with HF. ADHERE computes the BUN, SBP, and serum creatinine, which were found to be the most significant predictors of mortality following classification and regression tree analysis (CART) [5], whereas GWTG uses the SBP, BUN, sodium, age, heart rate, COPD history, and black race as independent variables to predict the studied outcome [3].

Statistical analysis

The demographic characteristics of the patients were reported as median ± interquartile range (IQR) for continuous variables and as frequencies for categorical variables. The normality of numerical variables was assessed prior to the imputation of missing data using the Shapiro-Wilk and Kolmogorov-Smirnov tests.

To compare differences between the deceased and survivor groups, the independent samples t-test was used for normally distributed variables, while the Mann-Whitney U test was applied for non-normally distributed variables. Categorical variables were analyzed by calculating absolute frequencies and percentages for each group, with the chi-square test employed to compare group frequencies. In all final analyses, p-values < 0.05 were considered statistically significant.

Model development and evaluation

Prior to selecting the final algorithm, we evaluated multiple supervised learning methods for structured data prediction, including logistic regression, Least Absolute Shrinkage and Selection Operator (LASSO), support vector classification (SVC), Gaussian naive Bayes, decision tree classifier, random forest classifier, multilayer perceptron classifier, k-nearest neighbors (KNN), and gradient boosting (XGBoost) [18]. Among these, XGBoost (XGB) demonstrated the highest area under the receiver operating characteristic curve (AUROC) during preliminary testing and was therefore selected, in line with prior studies utilizing the MIMIC database [19,20].

The XGB model was trained on the training set using manually defined hyperparameters, selected to optimize the balance between discrimination and generalizability. The algorithm’s built-in L1 and L2 regularization mechanisms penalize model complexity and help mitigate overfitting. Model performance was evaluated exclusively on the unseen test set, with the AUROC used as the primary metric [21].

All clinically relevant variables available in the dataset were initially included as input features, based on existing evidence and expert consensus. The XGB algorithm employs an embedded feature selection mechanism by calculating importance scores during training, enabling the model to automatically downweight or exclude less informative predictors. This internal selection process enhanced interpretability while avoiding pre-selection bias or the premature exclusion of potentially relevant variables. The relative importance of each feature was extracted and visualized to assess alignment with known clinical relevance.

**Figure 1:**
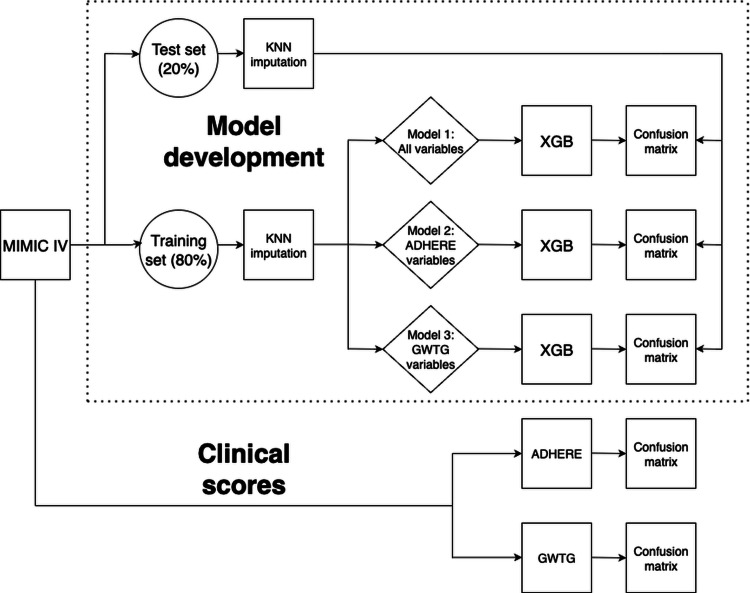
Model development flowchart Workflow of model development and evaluation. The Medical Information Mart for Intensive Care IV (MIMIC-IV) dataset was split into a training set (80%) and a test set (20%). Missing data were imputed using the k-nearest neighbors (KNN) algorithm. Three predictive models were developed using Extreme Gradient Boosting (XGB): Model 1 used all variables, Model 2 used the variables included in the Acute Decompensated Heart Failure National Registry (ADHERE) score, and Model 3 used the variables from the Get With The Guidelines-Heart Failure (GWTG-HF) score. The performance of the models was evaluated using confusion matrices. Clinical scores (ADHERE and GWTG-HF) were also calculated and assessed for comparison.

For comparison, we computed the ADHERE and GWTG-HF scores on the test set and evaluated their AUROC values. In all models, the target variable was in-hospital mortality. Additionally, we analyzed the main contributors to the XGB model’s performance using its internal feature importance metrics [18,21]. Figure 1 summarizes the overall modeling workflow.

## Results

The MIMIC-IV database contains 73,181 ICU admissions. We included 7,310 patients with heart failure (HF) who met the study's inclusion criteria. A total of 1,708 patients were excluded based on the exclusion criteria. Consequently, 5,602 patients were included in the final analysis. The patients were randomly assigned to either the derivation group (n = 4,481; 80%) or the validation group (n = 1,121; 20%).

We conducted a missing data analysis and excluded variables with more than 25% missing data. This step, performed for model development, resulted in the removal of the following features: NT-ProBNP, creatine kinase, height, lactate, neutrophils, basophils, lymphocytes, pH, and pCO2. For other missing variables, the k-nearest neighbors (KNN) imputation technique was applied [16].

The general characteristics of the participants are presented in Table 1. There were 346 in-hospital deaths, which accounted for 6.2% of the total patient population.

**Table 1:** General characteristics of studied population, and of patients who died and did not within the hospitalization ^a^ Number (%); ^b^ Indicates that vital signs were measured multiple times (every five minutes) during hospitalization, and the arithmetic mean of these measurements was used in the analysis. Data are presented as median ± interquartile range (IQR) for continuous variables and frequency (percentage) for categorical variables. SBP: systolic blood pressure; DBP: diastolic blood pressure; MBP: mean blood pressure; MCH: mean corpuscular hemoglobin; MCHC: mean corpuscular hemoglobin concentration; MCV: mean corpuscular volume; WBC: white blood cells; PT: prothrombin time; PTT: partial thromboplastin time; BUN: blood urea nitrogen; COPD: chronic obstructive pulmonary disease.

Variable	Overall	Dead	Alive	P-value
	(n = 5602)	(n = 346)	(n = 5256)	
Male sex^a ^	3047 (54.4)	194 (56.1)	2853 (54.3)	0.554
Age (years)	67.3 (55.3-79.4)	73.4 (61.4-82.2)	67 (54.9-79)	<0.001
Height(cm)	170 (161-178)	170 (160-178)	170 (163-178)	0.387
Black race^a^	681 (12.2)	34 (9.8)	647 (12.3)	0.171
Weight(kg)	78 (65.4-94.5)	75.6 (64.4-89.2)	78.3 (65.5-94.7)	0.016
SBP (mmHg)^b^	115.6 (106.2-128.4)	109.5 (101.8-118..6)	116.1 (106.6-129)	<0.001
DBP (mmHg)^b^	61.5 (54.9-69)	58.5 (51.5-65.5)	61.7 (55.2-69.2)	<0.001
MBP (mmHg)^b^	75.9 (69.5-83.5)	71.8 (66.5-78.9)	76.1 (69.7-83.9)	<0.001
Heart rate (bpm)^b^	105 (91-121)	113 (97-128)	105 (91-120)	<0.001
Respiratory rate (bpm)^b^	19.9 (17.2-22.9)	22.1 (19-25.3)	19.8 (17.2-22.7)	<0.001
Temperature (˚C)^b^	36.8 (36.6-37.2)	36.7 (36.3-37.2)	36.9 (36.6-37.2)	<0.001
Saturation of O_2_ (%)^b^	97.1 (95.4-98.5)	96.3 (94.3-98.3)	97.1 (95.5-98.5)	<0.001
Urine output (mL/day)	1500 (881.2-2330)	852 (330-1595)	1535 (923.5-2375)	<0.001
Hematocrit (%)	34.3 (29.9-39.3)	34.3 (29.8-39.2)	34.3 (29.9-39.3)	0.872
MCH (pg)	30.5 (28.9-32)	30.9 (29.3-32.5)	30.5 (28.8-32)	0.002
MCV (fL)	92 (88-97)	95 (89.8-101)	92 (88-97)	<0.001
WBC (10³/mm³)	12.4 (8.7-17.2)	14.4 (9.8-21.1)	12.2 (8.7-16.9)	<0.001
Platelets (10³/mm³)	227 (161-308)	198 (128-293)	228 (164-308)	<0.001
Neutrophils (10³/mm³)	9.5 (6.1-14.1)	11.1 (6.4-17.5)	9.4 (6.1-13.9)	<0.001
Basophils (10³/mm³)	0 (0-0)	0 (0-0)	0 (0-0)	<0.001
Lymphocytes (10³/mm³)	1.1 (0.7-1.7)	0.9 (0.5-1.8)	1.1 (0.7-1.7)	0.016
BUN (mg/dL)	25 (16-42)	36 (23-55)	25 (16-41)	<0.001
Creatinine (mg/dL)	1.2 (0.8-2)	1.6 (1.1-2.5)	1.2 (0.8-1.9)	<0.001
Potassium (mEq/L)	4.4 (4-4.9)	4.6 (4.1-5.4)	4.4 (4-4.9)	<0.001
Sodium (mEq/L)	140 (137-143)	140 (137-143)	140 (137-143)	0.648
Calcium (mg/dL)	8.6 (8.1-9)	8.5 (7.9-9.1)	8.6 (8.1-9)	0.165
Anion gap (mEq/L)	16 (14-19)	18 (15-22.8)	16 (14-19)	<0.001
pH	7.3 (7.2-7.4)	7.3 (7.1-7.4)	7.3 (7.3-7.4)	<0.001
Bicarbonate (mEq/L)	23 (19-26)	20 (15-24)	23(19-26)	<0.001
Magnesium (mg/dL)	1.9 (1.7-2.1)	1.9 (1.7-2.1)	1.9 (1.7-2.1)	0.385
Lactate (mmol/L)	1.8 (1.2-3)	3.3 (1.7-6.0)	1.7 (1.2-2.8)	<0.001
Glucose (mg/dL)	150.0 (121-200)	171 (130.5-247.5)	149 (120-197.8)	<0.001
NT-proBNP (pg/mL)	2976 (1017-9540.5)	5018 (1369.2-14698)	2952(1005-9124)	0.127
COPD^a^	598 (10)	66 (19.1)	532 (10.1)	<0.001
Diabetes^a^	449 (8.0)	53 (15.3)	396 (7.5)	<0.001
Renal disease^a^	320 (5.7)	41 (11.8)	279 (5.3)	<0.001
Myocardial infarction^a^	242 (4.3)	29 (8.4)	213 (4.1)	<0.001
Peripheral vascular disease^a^	140 (2.5)	21 (6.1)	119 (2.3)	<0.001
Cerebrovascular disease^a^	284 (5.1)	33 (9.5)	251 (4.8)	<0.001

Performance comparison

**Figure 2:**
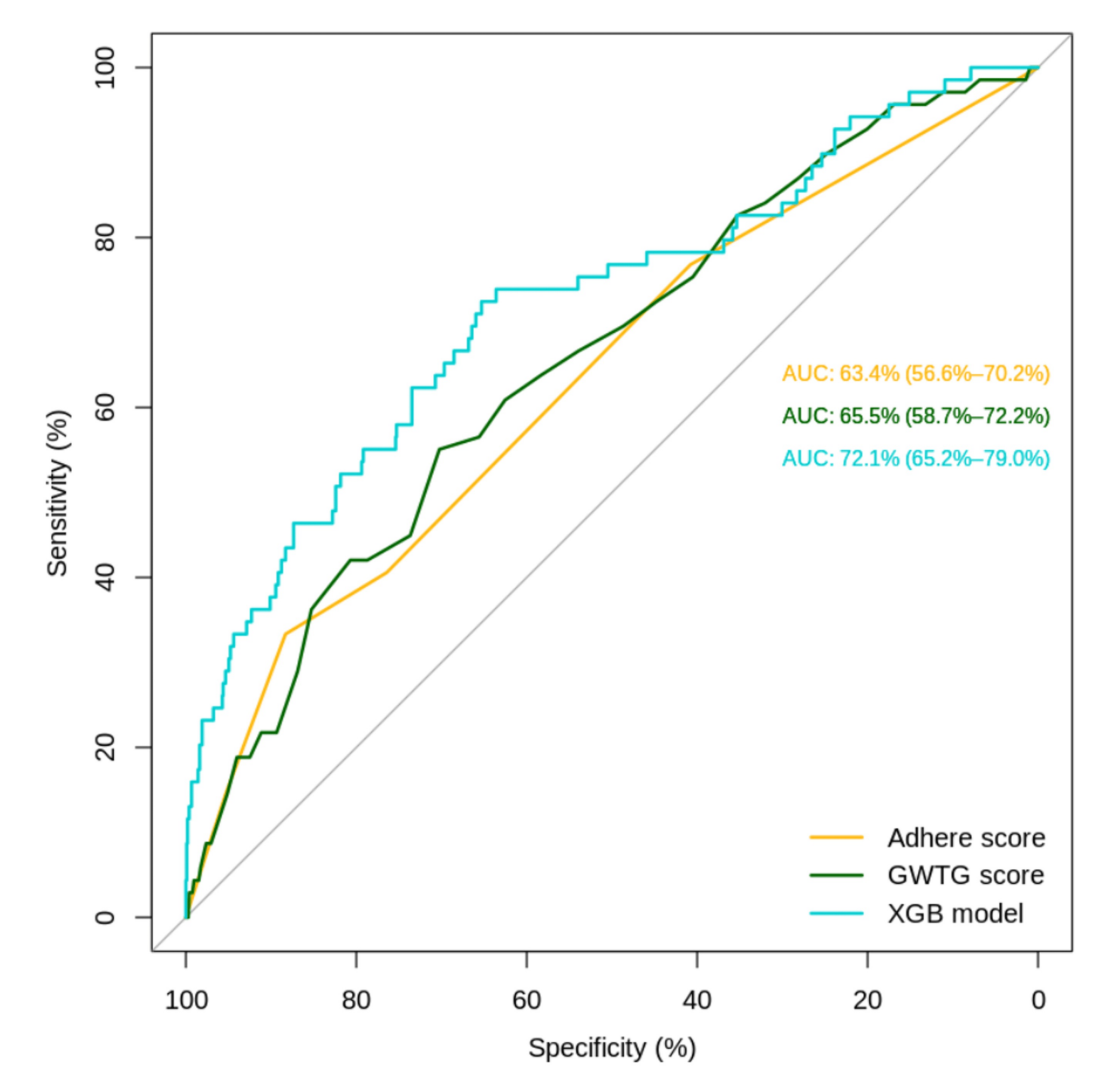
Receiver Operating Characteristic Curves for Mortality Prediction Models Comparison of receiver operating characteristic (ROC) curves for the predictive performance of the Extreme Gradient Boosting (XGB) model, Acute Decompensated Heart Failure National Registry (ADHERE) score, and Get With The Guidelines-Heart Failure (GWTG-HF) score for in-hospital mortality in patients with heart failure.

The performance of the XGB models was compared with that of the GWTG and ADHERE scores (Table 2). Table 3 presents the comparison values between the score performances. The XGB model using all variables achieved an AUROC of 72.11% (95% CI: 65.23%-79%), while the ADHERE score showed a performance of 63.39% (95% CI: 56.56%-70.21%) and the GWTG score achieved 65.47% (95% CI: 58.75%-72.19%) (Figure 2). The AUROC of the XGB model was significantly higher than that of the ADHERE model (p-value = 0.02). The difference between the AUROCs of the GWTG and XGB models did not reach statistical significance (p-value = 0.08), although the AUROC of the XGB model was higher.

**Table 2:** GWTG-HF and ADHERE scores for patients who died and survivors ^a^ Mean ± SD; ^b^ Number (%) GWTG-HF: Get With the Guidelines-Heart Failure; ADHERE: Acute Decompensated Heart Failure National Registry

Score / Risk Category	Overall (n = 5602)		Dead (n = 346)	Alive (n = 5256)	P-value
GWTG-HF^a^	45.0 ± 8.6	50.6 ± 8.4		44.6 ± 8.5	<0.001
ADHERE^b^					
High risk	355 (6.3)	50 (14.5)		305 (5.8)	<0.001
Intermediate risk	2994 (53.5)	218 (63.0)		2776 (52.8)	<0.001
Low risk	2253 (40.2)	78 (22.5)		2175 (41.4)	<0.001

**Table 3:** Comparison of the Area Under the ROC Curve (AUC) Comparison of the predictive performance of the models using the area under the ROC curve. AUC: area under the curve; CI: confidence interval; ADHERE: Acute Decompensated Heart Failure National Registry; GWTG-HF: Get With The Guidelines – Heart Failure; XGB: Extreme Gradient Boosting.

Models	AUC	CI 95%		Comparison	p-value
Adhere score	63.39%	56.56% - 70.21%		Adhere score vs GWTG-HF score	0.48
GWTG-HF score	65.47%	58.75% - 72.19%		Adhere score vs XGB all variables	0.02
XGB model with all variables	72.11%	65.23% - 79.00%		GWTG score vs XGB all variables	0.08

**Figure 3:**
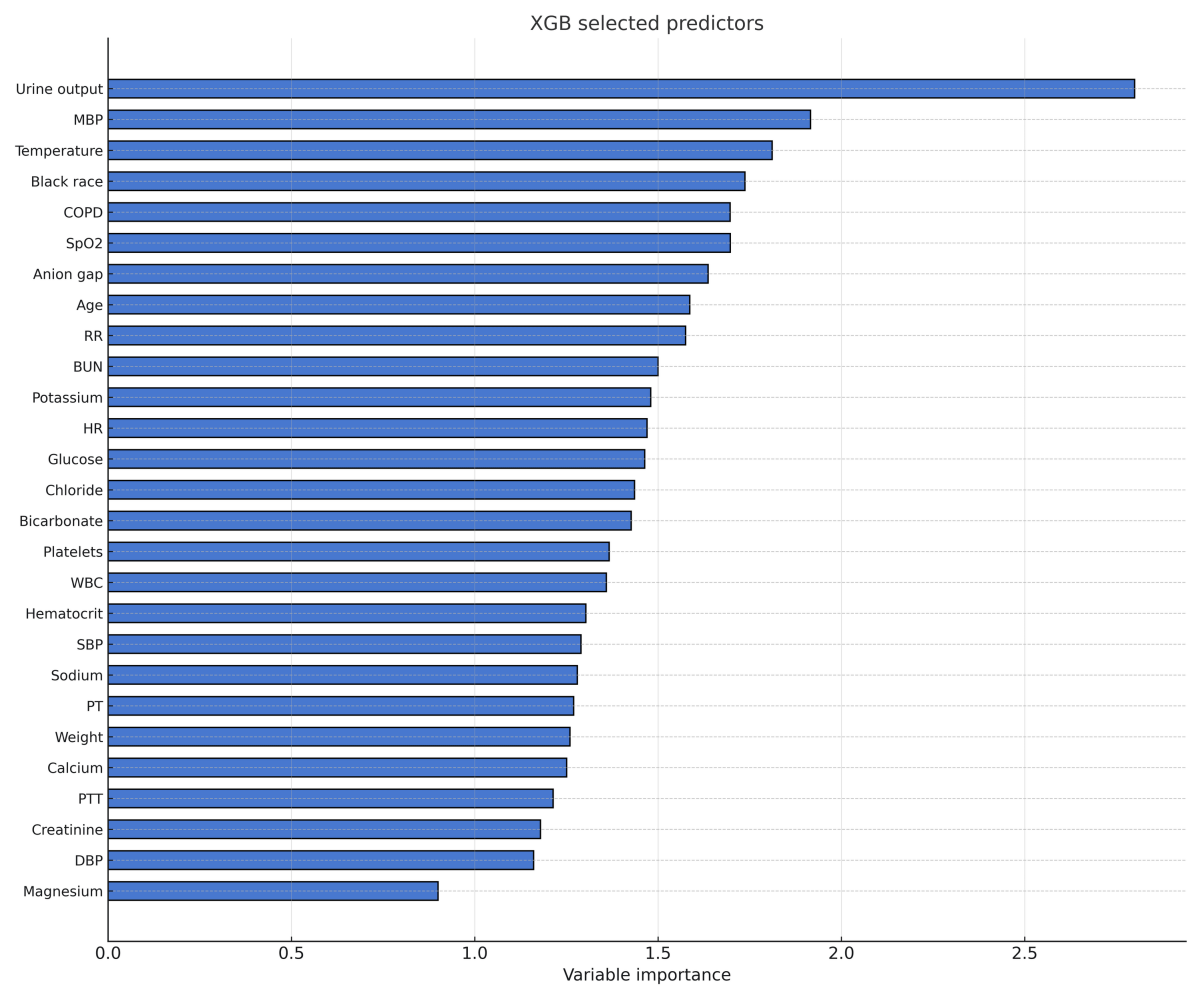
Predictors selected by XGB The figure shows the relative importance of each predictor variable in the Extreme Gradient Boosting (XGB) model. MBP: mean blood pressure; COPD: chronic obstructive pulmonary disease; SpO₂: peripheral oxygen saturation; RR: respiratory rate; BUN: blood urea nitrogen; HR: heart rate; WBC: white blood cells; SBP: systolic blood pressure; PT: prothrombin time; PTT: partial thromboplastin time; DBP: diastolic blood pressure.

The identification of predictive variables for risk assessment in patients with HF is critical for enhancing patient care. In our study, by employing the XGB algorithm, we found that the urine output, MBP, and temperature emerged as significant predictors of risk and were prioritized in descending order of importance. Figure 3 shows the most significant variables.

## Discussion

Our results revealed that the performance of our XGB-based model was superior to that of the GWTG and ADHERE scores, with statistical significance observed in the comparison between XGB and ADHERE. The analysis identified independent determinants of in-hospital mortality in a cohort of ICU-admitted heart failure patients, with the main predictive variables being urinary output and mean arterial pressure (MAP). The resulting model holds promise for enhancing clinical decision-making in the management of heart failure patients.

Despite significant advancements in pharmacological and nonpharmacological treatments, the prognosis of individuals with HF remains unfavorable [1,22,23]. The timing of interventions, particularly in the immediate post-deterioration phase after HF exacerbation, significantly influences the outcomes, with a heightened risk of readmission and early mortality. Rapid and intensive interventions during this critical period offer the potential for better results, thereby potentially reducing premature readmissions and mortality. The identification of high-risk patient groups allows for early multidisciplinary approaches, which improve decompensation factor recognition and treatment adherence, and ultimately decrease the hospitalization duration, morbidity, mortality, and resource utilization associated with HF [22,24].

GWTG-HF and ADHERE are classical scores used for risk prediction in hospitalized HF patients [5,6]. However, their performance was inferior to that of our XGB-based model. These scores were developed over a decade ago and lack a structure that enables continuous improvements in prediction performance and personalization. They were created using statistical methods that fail to capture important multidimensional correlations needed to estimate prognosis [9] and were designed based on specific populations, which hinders their applicability in diverse contexts.

Other studies have also compared the performance of machine-learning techniques using risk scores to predict the risk in patients with HF. A retrospective cohort study conducted by Li et al. aimed to develop and validate a predictive model for in-hospital mortality among ICU-admitted HF patients using data from the MIMIC-III database. Notably, the XGB and LASSO regression models exhibited superior predictive capabilities compared with the GWTG-HF risk score model (AUROC = 0.8416 vs. 0.7747). The selected XGB model, presented in the form of a model, provides a concise and efficient tool for forecasting in-hospital mortality in ICU-admitted patients with HF, thereby aiding in informed clinical decision-making [19]. In this study, a better AUROC than ours was achieved, likely due to the larger number of included patients (13,389) and the use of echocardiographic data.

Chen et al. also applied machine learning techniques using the MIMIC-IV database (training group - 20,878 patients) combined with the eICU database (validation group - 15,478 patients) to develop a mortality risk prediction tool for HF. Once again, XGB (AUROC: 0.771) demonstrated the best performance compared to GWTG and logistic regression [25]. The combination of databases provided the study with a representative number of patients. The XGB model has been consistently highlighted in the literature as a concise and efficient tool for predicting in-hospital mortality in ICU-admitted HF patients, with the potential to assist in informed clinical decision-making [19,25].

In our study, using the XGB algorithm, BUN and blood pressure emerged as strong contributors to the in-hospital mortality risk prediction model, similar to the GWTG-HF [6] and ADHERE [5] scores. We also identified urinary output as a significant risk predictor. Clinically, we were able to draw a parallel between the importance of urinary output and cardiorenal syndrome. This syndrome involves complex bidirectional pathophysiological pathways related to cardiac and renal dysfunctions. Venous congestion, reduced arterial filling, neurohormonal activation, inflammation, and endothelial dysfunction contribute to this syndrome [26]. Li et al. also reported urinary output as one of the most important variables selected by XGB [19]. Exploring urinary output as a predictive variable presents a promising opportunity, as it is an easily accessible measure. In our database, it had a low percentage of missing data (3.6%).

Despite the use of a comprehensive dataset, this study is subject to the inherent limitations of retrospective cohort designs. These include incomplete data, potential calibration biases, and the difficulty of fully controlling for confounding variables. Additionally, the generalizability of our findings to other populations remains uncertain. To enhance the robustness of our analysis, we refined the inclusion criteria to better reflect real-world scenarios and applied data imputation techniques to minimize bias from missing values. The class imbalance (6.2% in-hospital mortality) may have influenced probability thresholds and model generalization. Although stratified sampling and XGBoost’s internal mechanisms helped mitigate this issue, future studies should explore threshold tuning and cost-sensitive learning approaches. While the XGB model achieved a higher AUROC than both GWTG-HF and ADHERE, the difference compared to GWTG-HF was not statistically significant (p = 0.08), warranting cautious interpretation. Finally, model calibration was not assessed in this version. As accurate risk estimation is crucial for clinical utility, future work should include calibration plots and metrics such as the Brier score to evaluate the agreement between predicted and observed outcomes.

## Conclusions

In this study, we developed a machine learning model based on the XGB algorithm to predict in-hospital mortality among heart failure patients admitted to cardiac intensive care units. The model outperformed the ADHERE score and showed a slightly higher AUROC than GWTG-HF, although the difference was not statistically significant. Urinary output and mean arterial pressure emerged as key predictors, reinforcing their importance in clinical decision-making. Despite moderate discriminative performance, the model demonstrates promising potential for improving risk stratification. To enhance its clinical applicability, future studies should focus on external validation, calibration assessment, and the creation of a user-friendly interface to support implementation in real-world settings.

## Appendices

The research data are available at: MIMIC-IV v2.2 (physionet.org).

The code used to perform all steps is available at https://github.com/pedrogemal/mortality-prediction-in-hf.
